# Downregulation of HSP60 disrupts mitochondrial proteostasis to promote tumorigenesis and progression in clear cell renal cell carcinoma

**DOI:** 10.18632/oncotarget.9615

**Published:** 2016-05-26

**Authors:** Haiping Tang, Yuling Chen, Xiaohui Liu, Shiyu Wang, Yang Lv, Di Wu, Qingtao Wang, Minkui Luo, Haiteng Deng

**Affiliations:** ^1^ MOE Key Laboratory of Bioinformatics, School of Life Sciences, Tsinghua University, Beijing, China; ^2^ Center of Nephrology, The General Hospital of the PLA, Beijing, China; ^3^ Beijing Chaoyang Hospital, Capital Medical University, Beijing, China; ^4^ Molecular Pharmacology and Chemistry Program, Memorial Sloan-Kettering Cancer Center, New York, NY, USA

**Keywords:** renal cancer, HSP60, proteostasis, reactive oxygen species, tumorigenesis

## Abstract

In the present study, we demonstrate that HSP60 is unequivocally downregulated in clear cell renal cell carcinoma (ccRCC) tissues compared to pericarcinous tissues. Overexpression of HSP60 in ccRCC cancer cells suppresses cell growth. HSP60 knockdown increases cell growth and proliferation in both cell culture and nude mice xenografts, and drives cells to undergo epithelial to mesenchymal transition (EMT). Our results propose that HSP60 silencing disrupts the integrity of the respiratory complex I and triggers the excessive ROS production, which promotes tumor progression in the following aspects: (1) ROS activates the AMPK pathway that promotes acquisition of the Warburg phenotype in HSP60-KN cells; (2) ROS generated by HSP60 knockdown or by rotenone inhibition drives cells to undergo EMT; and (3) the high level of ROS may also fragment the Fe-S clusters that up regulates ADHFe1 expression and the 2-hydroxygluterate (2-HG) production leading to changes in DNA methylation. These results suggest that the high level of ROS is needed for tumorigenesis and progression in tumors with the low HSP60 expression and HSP60 is a potential diagnostic biomarker as well as a therapeutic target in ccRCC.

## INTRODUCTION

Mitochondrial proteostasis is regulated by the proteostasis network that plays a crucial role in maintenance of conformation, concentration, localization, and interactions of individual protein in the mitochondrial proteome [1]. Deterioration of proteostasis network has been implicated in aging and aging-associated diseases [2, 3]. HSP60 is one of the major classes of ATP-dependent chaperones in mitochondria and is one of the most conserved proteins from bacteria to mammals. HSP60 has been implicated in many complex diseases including neurodegenerative disorders, atherosclerosis and heart disease, as well as multiple inflammatory diseases [4–6]. Functions of HSP60 in cancer have been extensively studied showing that HSP60 elicits both pro-survival and pro-apoptotic functions in tumors. On one hand, HSP60 was found to enhance tumor cell growth, to suppress stress-induced apoptosis and to promote tumorigenesis and metastasis [7–9]. The anti-apoptotic function of HSP60 was attributable to the binding of HSP60 with cyclophilin D (CypD) that regulated the mitochondrial permeability transition pore [10]. Consequently, high levels of HSP60 were present in colorectal carcinogenesis, ovarian cancer, and prostate cancers [11–13]. On the other hand, down-regulation of HSP60 was reported in lung cancer and bladder cancer [14–15]. These results indicate that HSP60 executes the tumor-type dependent function and its precise role in tumorigenesis needs to be elucidated in the context of a specific cancer.

Clear Cell Renal Cell Carcinoma (ccRCC) is the most common histological subtype of renal cancer [16]. The development of ccRCC is mainly attributed to the mutation of the von Hippel–Lindau gene that stabilizes hypoxia-inducible factors, leading to accumulation of glycogens and fat in tumor cells. Mutations in mTOR and PI3K render ccRCC as a metabolic disease [17] as confirmed by the low level of fructose-1,6-bisphosphatase (FBP1) in most ccRCC patients that antagonized glycolytic flux [18]. A more recent study found that ccRCC cells were sensitive to glycolytic inhibition due to minimal mitochondrial respiratory capacity [19]. This supports the long standing hypothesis that mitochondrial dysfunction is involved in tumorigenesis. As a major chaperone for maintaining mitochondrial proteostasis, HSP60 expression is expected to be low in ccRCC. However, reports on HSP60 expression in ccRCC are rather controversial. Early proteomic studies with 2D gel electrophoresis and other mass spectrometric methods show that the HSP60 expression is low in ccRCC compared to normal kidney tissues [20–23], which is not confirmed in recent proteomic studies [24–25].

In the present study, we have examined HSP60 levels in ccRCC and adjacent kidney tissues from the same patient. Our results demonstrate that the HSP60 expression is unequivocally lower in cancer tissues than that in the associated pericarcinous tissues. Using HSP60 knockdown cells, we demonstrate that HSP60 silencing promotes the Warburg effect and drives cells to undergo EMT via disruption of mitochondrial proteostasis. Our results propose that HSP60 silencing promotes tumor progression of ccRCC and HSP60 is a potential biomarker for ccRCC diagnosis.

## RESULTS

### Downregulation of HSP60 expression in ccRCC compared to associated pericarcinous tissues

**Figure 1:**
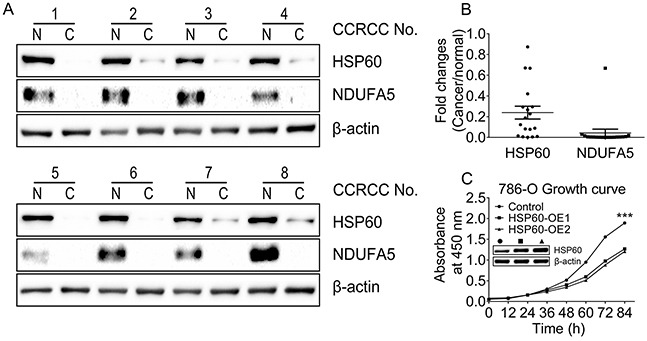
Downregulation of HSP60 and NDUFA5 in ccRCC compared to associated pericarcinous tissues **A.** Western blotting images of the expression levels of HSP60 and NDUFA5 in 8 of 18 paired ccRCC lesions and associated pericarcinous tissues; **B.** the gray scale analysis of HSP60 and NDUFA5 in 18 paired ccRCC lesions compared to associated pericarcinous tissues; and **C.** Growth curves of HSP60-overexpressing and the control cells showing that HSP60 overexpression slows down cell proliferation rates in 786-O cells.

A total of 18 paired ccRCC lesions and associated pericarcinous tissues were analyzed in the present study. Briefly, equal amounts of proteins extracted from each pair of tissue samples were analyzed by western blotting (Figure 1(A)) showing the HSP60 and NADH dehydrogenase (ubiquinone) 1 alpha subcomplex 5 (NDUFA5) expressions in 8 pairs of samples. The gray scale analysis of western blot data for all 18 pairs of samples revealed that the average HSP60 and NDUFA5 expression levels were 4 fold and 10 fold lower in ccRCC than those in the pericarcinous tissues, respectively (Figure 1(B)). This demonstrated that both HSP60 and NDUFA5 were decreased in ccRCC, suggesting that HSP60 silencing was important for tumorigenesis and progression. Furthermore, HSP60 overexpression in ccRCC cell line 786-O resulted in a markedly decrease in proliferation rate (Figure 1(C)), which reinforced that the low HSP60 expression was needed in ccRCC.

### Characterization of HSP60-knockdown Cells

**Figure 2:**
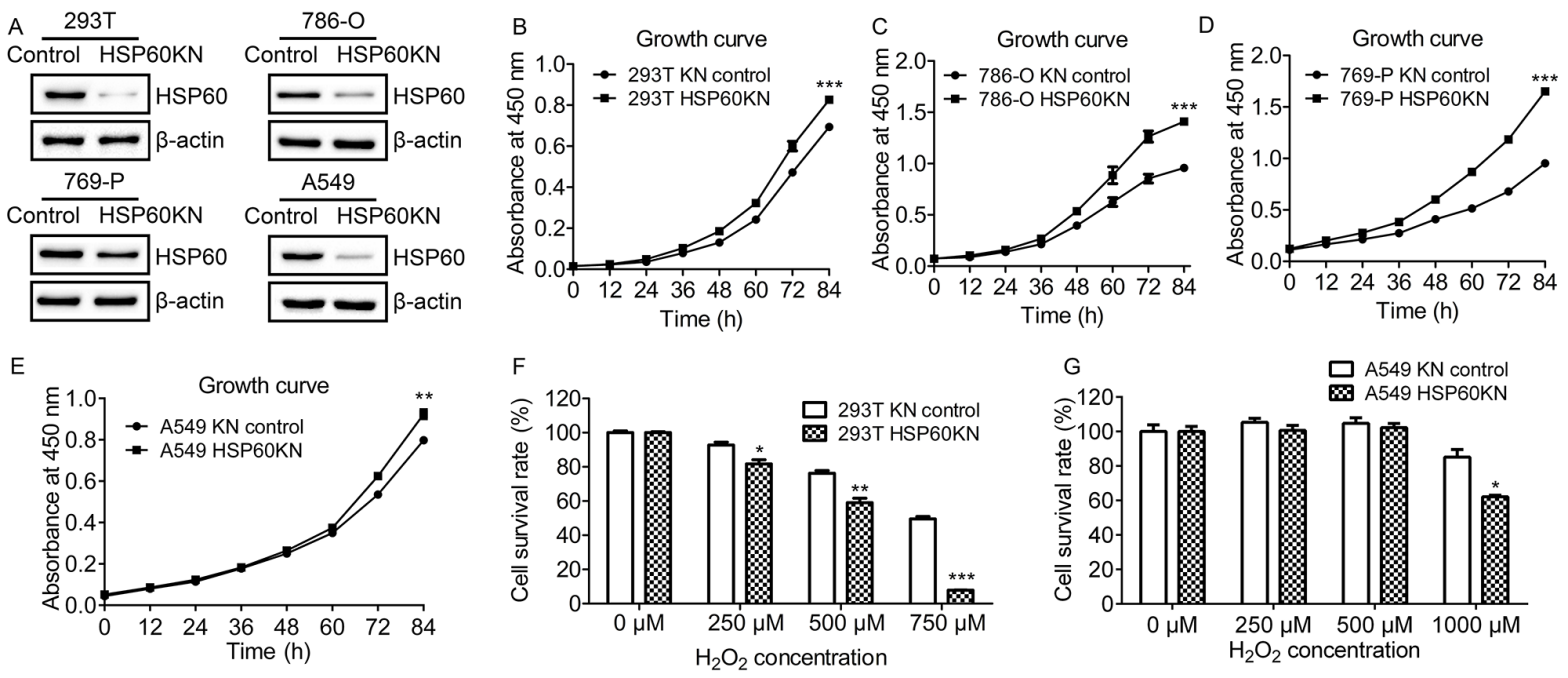
Characterization of HSP60-knockdown cells **A.** Western blotting images of HSP60 in the control and HSP60-KN cells showing that the expression of HSP60 was decreased in HSP60-KN cells compared to control cell lines. **B.** Growth curve of HSP60-KN-293T and the control cells; **C.** Growth curve of HSP60-KN-786-O and the control cells; **D.** Growth curve of HSP60-KN-769-P and the control cells; **E.** Growth curve of HSP60-KN-A549 and the control cells; **F-G.** Survival rate of HSP60-KN-293T, HSP60-KN-A549 and their control cells treated with different concentration of H_2_O_2_. Data were analyzed using student's t test. *p<0.05, **p<0.01 and *** p< 0.001. *p < 0.05 is considered statistically significant. Error bars represent ±SEM.

To understand the role of HSP60 in tumorigenesis and progression of ccRCC, HSP60-directed shRNAs were selected and used to silence HSP60 in multiple cell lines including 293T, A549, 786-O and 769-P cells. The cells transfected with a non-silencing scrambled control shRNA was used as the control. The expression of HSP60 in these cells was examined by western blotting (Figure 2(A)), confirming that the expression levels of HSP60 in HSP60-KN cells was lower than those in control cells.

HSP60 knockdown promoted growth and proliferation of HSP60-KN cells as compared to control cells (Figure 2(B)-(E)). To determine the susceptibility of HSP60-KN cells to oxidative stress, cells were treated with various concentrations of hydrogen peroxide for 12 h. Cell viability was measured using CCK-8 assay. The effects of hydrogen peroxide were represented as the percentage of viable cells after 12 h treatment (Figure 2(F) and (G)). When cells were treated with 750 μM H_2_O_2_ for 12 h, percentages of viable cells were 55% and 10% for control and HSP60-KN-293T cells, respectively (Figure 2(F)). Similarly, when A549 and HSP60-KN-A549 cells were treated with 1000 μM H_2_O_2_ for 12 h, percentages of viable cells were 80% and 60%, respectively (Figure 2(G)). This declares that HSP60-KN cells are more sensitive to H_2_O_2_ treatment and it also shows that ROS generating reagents such as gossypol are more efficient to eliminate HSP60 knockdown cells (Supplementary Figure S1).

**Figure 3:**
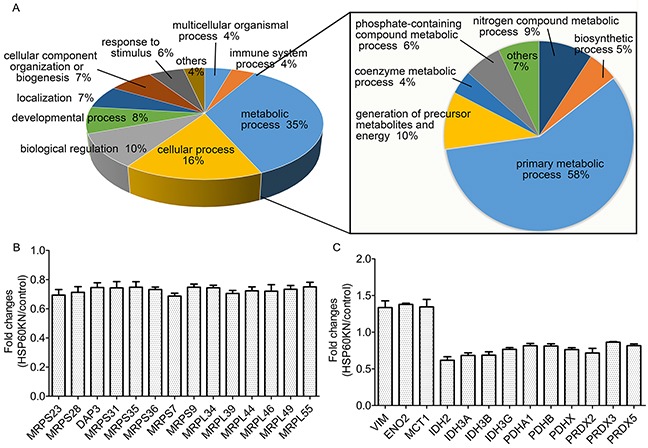
Proteomic analysis of differentially expressed proteins between HSP60-KN-293T and control cells **A.** GO analysis of the down-regulated proteins in HSP60-KN-293T cells compared to the control cells; **B.** Graphical representation of TMT ratios for subunits of mitochondrial ribosome in HSP60-KN-293T cells compared to the control cells; **C.** Expression levels of proteins in TCA cycle and antioxidant were downregulated whereas proteins associated with glycolysis and vimentin were upregulated in HSP60-KN-293T cells.

Next, proteomic analysis was carried out on HSP60-KN-293T and control cells in biological triplicates. Equal amounts of proteins from both cells were in solution digested, labeled with Tandem Mass Tag (TMT) reagents and mixed. The generated tryptic peptides were fractionated using off-line HPLC and each fraction were further analyzed by nano-LC-MS/MS. Differentially expressed proteins were identified and quantified using TMT-based quantitation. We identified 9194 proteins in three biological replicates and the false-positive rate was estimated to be less than 1%. Based on the average reporter ion ratios (>1.5 or <0.67), 172 proteins were found to be differentially expressed between HSP60-KN-293T and control cells, in which 151 proteins were down-regulated and 21 were up-regulated (Supplementary Table S1 and S2). In order to understand the biological relevance of the differentially expressed proteins, the Gene Ontology (GO) was used to cluster down-regulated proteins according to their associated biological processes. The annotations of gene lists are summarized via a pie plot based on the biological process from Panther (www.pantherdb.org/) as shown in Figure 3(A). One hundred and fifty one down-regulated proteins participate in a variety of cellular processes including metabolic processes, cellular process, and biological regulation process. The primary metabolic process is the dominant difference between HSP60-KN-293T and the control cells. About 30% of all differentially expressed proteins are classified as mitochondrial proteins, indicating that these proteins are potential client proteins or downstream targets of HSP60, in which 14 mitochondrial ribosomal proteins are down regulated in HSP60-KN-293T cells (Figure 3(B)), which was confirmed by the qPCR analysis of mRNAs of the selected mitochondrial ribosome subunit genes (Supplementary Figure S2). We also noticed that pyruvate dehydrogenase E1 component subunit alpha (PDHA1) were down-regulated (Figure 3(C)) which was confirmed by western blot analysis (Figure 5(A)), suggesting that the flow from glycolysis to TCA cycle was hindered in HSP60-KN-293T cells. Consequently, IDH2 in TCA cycle was downregulated whereas proteins enolase 2 (ENO2) and monocarboxylate transporter 1 (MCT1) associated with glycolysis were increased in HSP60-KN-293T cells. All these results indicate that HSP60-silencing disrupts mitochondrial proteostasis and contributes to mitochondrial dysfunction. Downregulation of antioxidant enzymes PRDX2, PRDX3, and PRDX5 contributes to the enhanced susceptibility to oxidative stress in HSP60-KN-293T cells (Figure 3(C)).

### HSP60 knockdown-mediated high level ROS production activates AMPK pathway in HSP60-KN cells

**Figure 4:**
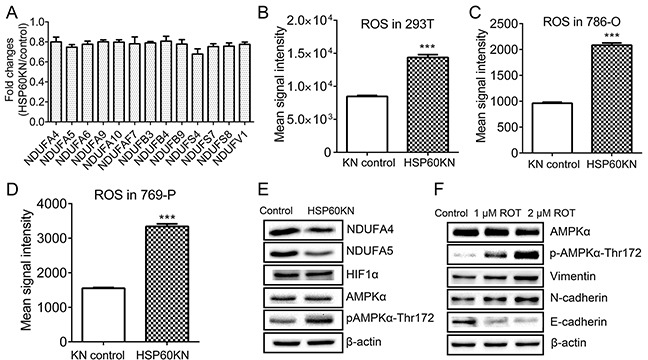
HSP60 knockdown disrupts complex I integrity to activate AMPK pathway **A.** Graphical representation of TMT ratios for subunits of respiratory Complex I in HSP60-KN-293T cells compared to the control cells; **B.** Graphical representation of ROS levels of HSP60-KN-293T cells compared to the control cells; **C.** Graphical representation of ROS levels of HSP60-KN-786-O cells compared to the control cells; **D.** Graphical representation of ROS levels of HSP60-KN-769-P cells compared to the control cells; **E.** Western blotting images of the selected proteins and phosphoproteins associated with complex I and AMPK pathway in HSP60-KN-293T cells; and **F.** Western blotting of expression levels of AMPK, E-cadherin, N-cadherin and vimentin in 293T cells which were treated with 1 μM and 2 μM rotenone for 24 h. With the treatment, E-cadherin was downregulated while vimentin and N-cadherin were up regulated in treated cells.

Thirteen subunits of respiratory complex I were down-regulated in HSP60-KN-293T cells (Figure 4(A)) as confirmed by western blotting of NADH dehydrogenase [ubiquinone] 1 alpha subcomplex subunit 4 and 5 (NDUFA4 and NDUFA5) in HSP60-KN-293T cells (Figure 4(E)), suggesting that disruption of respiratory complexes in ccRCC was caused by HSP60 silencing. Quantitative proteomic analysis of the immunoprecipitated HSP60 complex identified that HSP60 bound with multiple subunits of complex I including NDUFA3, NDUFA4, NDUFA5, NDUFB9, NDUFAF3, NDUFAF4, NDUFA8, NDUFA9, NDUFA11, NDUFB6, and NDUFB10 (Supplementary Table S3). The binding of HSP60 with NDUFA4, NDUFA5, and NDUFB9 was further confirmed by western blot analysis of the immunoprecipitated HSP60 complex (Supplementary Figure S3). Respiratory complex I is the major site for ROS production and the down-regulation of its subunits may cause dysfunction of the complex I to generate excessive ROS [26–27]. The cellular ROS levels were measured with CellROX® Deep Red kit and it showed that HSP60-KN cells exhibited much stronger fluorescence with increased ROS levels in 293T, 786-O and 769-P cells, respectively (Figure 4(B)-4(D)).

To examine if ROS activates the downstream signaling pathways [28–29], western blotting was used to probe changes in HIF1 and AMPK (Figure 4(E)). As a result, the expression of both HIF-1 and AMPK alpha subunit did not display significant difference between HSP60-KN-293T and control cells whereas the phosphorylation level of AMPKα at T172 was elevated (Figure 4(E)). Similar results were observed in HSP60-KN-786-O cells (Supplementary Figure S4). The increase of AMPKα phosphorylation indicated that AMPK was activated possibly by ROS [30]. To test our hypothesis, 293T cells were treated with rotenone, an inhibitor of respiratory complex I. Indeed, rotenone treatment increased AMPKα phosphorylation at T172 (Figure 4(F)). Western blotting also showed that expressions of EMT markers were increased in rotenone-treated cells.

### HSP60 knockdown enhances glycolysis leading to Warburg metabolic phenotype

**Figure 5:**
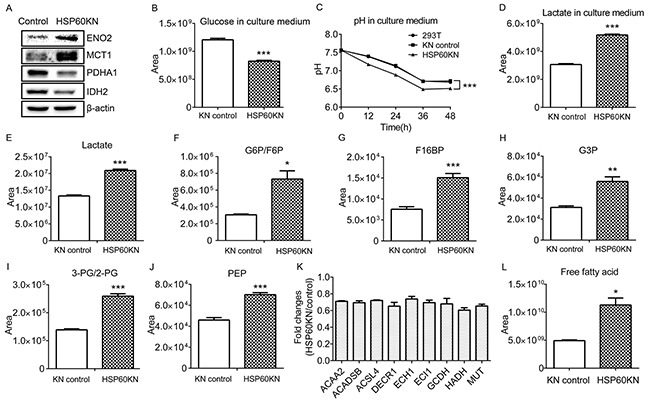
HSP60 knockdown enhances glycolysis via AMPK pathway **A.** Western blotting images of ENO2, MCT1, PDHA1, and IDH2 in HSP60-KN-293T cells compared to the control cells; **B.** Graphical representation of the relative levels of glucose in cell culture medium; **C.** The pH value of the cell culture medium of HSP60-KN-293T cells and the control cells; **D.** Graphical representation of the relative concentration of lactate in cell culture medium. **E-J.** Graphical representations of relative concentrations of lactate; G6P/F6P; F16BP; G3P; 3-PG/2-PG; and PEP in HSP60-KN-293T cells compared to the control cells; **K.** Expression levels of proteins in fatty acid β-oxidation were downregulated in HSP60-KN-293T cells; and **L.** Levels of free fatty acid in HSP60-KN-293T cells are higher than that in the control cells. Data were analyzed using student's t test. *p<0.05, **p<0.01 and *** p< 0.001. Error bars represent ±SEM.

It is known that AMPK enables the enhancement of glycolysis [31]. Western blotting showed that glycolysis associated proteins ENO2 and MCT1 expressions were increased in HSP60-KN-293T cells (Figure 5(A)), consistent with proteomic results. Using metabolomic profiling, it was observed that glucose levels in the culture medium of the HSP60-knockdown cells were lower than those of the control cells (Figure 5(B)), and the pH value of the cell medium was 0.3 units lower as well (Figure 5(C)), indicating that HSP60-KN-293T cells secreted a higher amount of lactate into the medium. Indeed, lactate concentrations were elevated in both HSP60-KN-293T cells and their medium compared to controls (Figure 5(D-E)), which was consistent to the AMPK mediated up-regulation of MCT1 (Figure 5(A)) [32]. To further confirm that glycolysis was enhanced by HSP60 knockdown, the steady state levels of metabolites of glycolysis were measured showing that all glycolytic intermediates were elevated in HSP60-KN-293T cells (Figure 5(F)-(J). Similar results were observed in the HSP60-KN-786-O cells and HSP60-KN-769-P cells (Supplementary Figure S5). We also identified that nine proteins in fatty acid β-oxidation pathway were downregulated (Figure 5(K)), which is consistent with metabolomic analysis showing that levels of free fatty acids were two folds higher in HSP60-KN-293T cells than those in the control cells (Figure 5(L)).

### HSP60 Knockdown enhances production of 2-OG and 2-HG in HSP60-KN-293T cells

**Figure 6:**
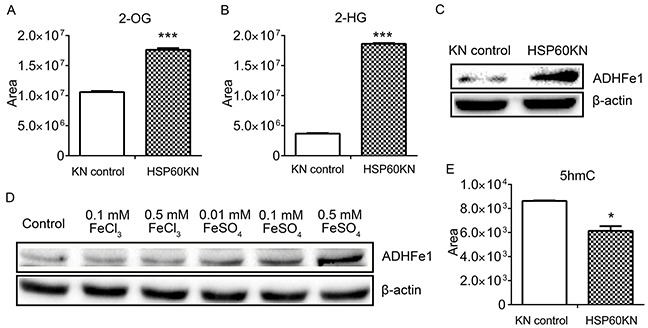
HSP60 knockdown increases the cellular concentration of 2-HG **A.** The level of 2-OG and **B.** the levels of 2-HG in HSP60-KN-293T cells compared to the control cells. **C.** Western blotting images showing expression levels of ADHFe1 in HSP60-KN-293T cells compared to the control cells. **D.** Western blotting images showing changes in the expression of ADHFe1 in the control cells treated with different concentration of FeCl_3_ or FeSO_4_ for 12 h. **E.** Levels of 5hmC were decreased in HSP60-KN-293T cells compared to the control cells. Data were analyzed using student's t test. *p<0.05, **p<0.01 and *** p< 0.001. Error bars represent ±SEM.

Western blot analysis showed the reduction of IDH2 in HSP60-KN cells that may affect metabolites in TCA cycle (Figure 5(A)). Metabolomic analysis revealed that most metabolites in TCA cycle were not affected by HSP60 knockdown except 2-oxoglutarate (2-OG) and 2-hydroxyglutarate (2-HG) (Figure 6(A)-6(B)). The cellular level of 2-OG in HSP60-KN-293T cells was approximately twice of that in control cells, while 2-HG level in HSP60-KN-293T was elevated by a factor of four. Similar results were observed in HSP60-KN-786-O cells and HSP60-KN-769-P cells (Supplementary Figure S5). This observation demonstrates formation of 2-HG was enhanced dramatically by HSP60 knockdown. It was known that IDH1 mutation is the major cause of 2-HG production from 2-OG [33]. In the present experiment, mutations of IDH1 were not found in both HSP60-KN-293T and the control cells, indicating alternative enzymes responsible for transformation of 2-OG to 2-HG. It is known that alcohol dehydrogenase iron-containing protein 1 (ADHFe1) catalyzes the reaction of hydroxybutanoate and 2-oxoglutarate to form acetoacetate and 2-HG [34]. In proof of this hypothesis, the expression of ADHFe1 was examined by western blotting and showed that ADHFe1 was up-regulated in HSP60-KN-293T cells (Figure 6(C)). ADHFe1 is an iron-containing protein and its expression may be regulated by labile Fe^2+^ ions. Treating the cells only with Fe^2+^ induced up-regulation of ADHFe1 (Figure 6(D)), suggesting the higher ROS level in HSP60-KN-293T cells attacked solvent-exposed [4Fe-4S] cluster to release liable Fe^2+^ ions that increased ADHFe1 expression. Furthermore, 2-HG as an inhibitor of 2-OG-dependent dioxygenases may inhibit activities of Ten-Eleven Translocation (TET) protein that catalyzes conversion of 5-methylcytosine (5mC) to 5-hydroxymethylcytosine (5hmC) [35]. The level of 5hmC was assessed by LC-MS/MS analysis showing it was lower in HSP60-KN-293T cells (Figure 6(E)).

### HSP60 knockdown activates epithelial-mesenchymal transition in 293T cells

**Figure 7:**
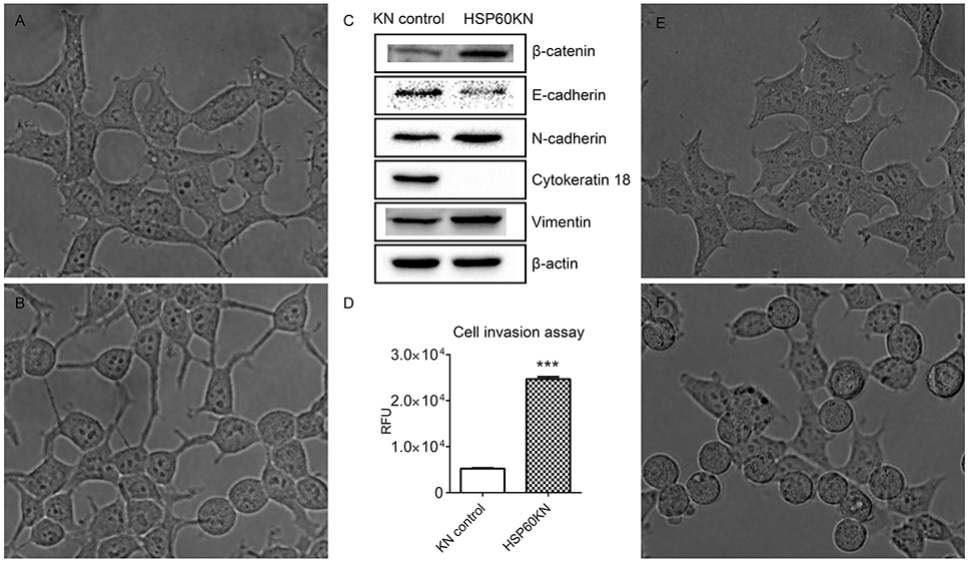
HSP60-KN-293T cells undergo the EMT process **A.** and **B.** represent the morphologies of the control cells and HSP60-KN-293T cells. **C.** Western blotting images of expression levels of β-catenin, E-cadherin, N-cadherin, cytokeratin 18 and vimentin in the control cells and HSP60-KN-293T cells. **D.** Cell invasion assay showing that HSP60-KN-293T cells displayed a higher migration rate than the control cells did. Data were analyzed using student's t test. *p<0.05, **p<0.01 and *** p< 0.001. Error bars represent ±SEM. **E.** and **F.** represent the morphologies of untreated 293T cells and 293T cells treated with 1 μM rotenone for 12 h.

In contrast with control cells, HSP60-KN-293T cells demonstrated a dramatic change of cell morphology, with transformation of the epithelial cells to a smaller and spindle-like morphology that bears the similar shape as the intermediate of EMT (Figure 7(A)-7(B)) [36]. The HSP60 knockdown mediated EMT phenotype was confirmed by western blotting of expressions of EMT markers in 293T cells (Figure 7(C)). Similarly, HSP60 knockdown also induced upregulations of EMT markers in 786-O cells (Supplementary Figure S4), presenting that HSP60-KN cells exhibited significantly higher expressions of the mesenchymal markers N-cadherin and vimentin, and the lower expressions of the epithelial markers E-cadherin and cytokeratin 18. Using cell invasion assay, HSP60-KN-293T cells were found to display a higher migration rate (Figure 7(D)). Thus, we demonstrate that HSP60-knockdown cells undergo the EMT process in 293T and 786-O cells. The role of ROS in HSP60-mediated EMT was also assessed by treating control cells with rotenone, resulting in the similar cell morphological changes as exhibited by HSP60 knockdown (Figure 7(E)-7(F)) as confirmed by western blotting of EMT markers (Figure 4(E)). It can be concluded that ROS is the driving force in HSP60-knockdown mediated EMT in 293T cells.

### HSP60-KN cells exhibited increased in vivo growth rate in nude mice compared to control cells

**Figure 8:**
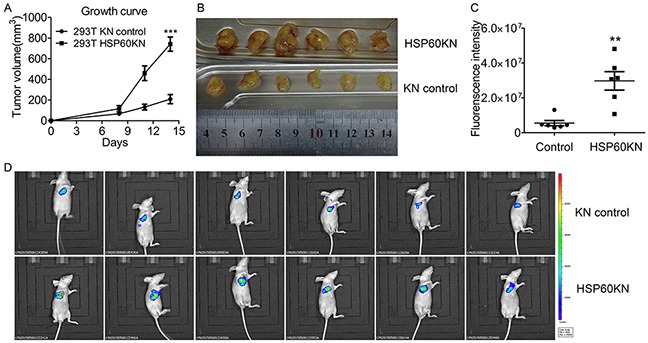
HSP60 knockdown promotes in vivo tumor growth in nude mice **A.** Growth curves of tumors produced by HSP60-KN-293T and the control cells which were injected subcutaneously into nude mice. The tumor volumes (mm^3^) were measured using digital calipers every 3 days after injection and calculated using the formula: π/6× length (mm) × width^2^ (mm). Data were analyzed using student's t test. *p<0.05, **p<0.01 and *** p< 0.001. Error bars represent ±SEM. **B.** Images of tumor samples harvested from mice 14 days after injection of HSP60-KN-293T or the control cells. **C.** Quantitation of the fluorescence intensities of the images. Data were analyzed using student's t test. *p<0.05, **p<0.01 and *** p< 0.001. Error bars represent ±SEM. **D.** Fluorescence images of tumors from mice injected with HSP60-KN-293T or the control cells.

In Petri dish, HSP60-KN-293T, HSP60-KN-786-O, HSP60-KN-769-P and HSP60-KN-A549 cells displayed the higher growth rates than the control cells, indicating that the low HSP60 expression is beneficial to cell growth. Injection of HSP60-KN-293T cells subcutaneously into 5-week-old immune-compromised mice gave rise to exponentially growing tumors. HSP60-KN-293T cells grew faster than the control cells shown in Figure 8(A)-8(B), as quantified by florescence imaging (Figure 8(C)-8(D)). Similar results were observed for HSP60-KN-A549 cells (Supplementary Figure S6). Xenograft experiment in nude mice demonstrates that HSP60-knockdown significantly accelerate tumor growth of 293T and A549 cells in nude mice, providing further evidence to demonstrate that HSP60 knockdown promotes cancer cell progression.

## DISCUSSION

Decreased mitochondrial respiratory capacity renders ccRCC as a mitochondrial disease [17–19]. This is supported by our finding that HSP60 expression is lower in ccRCC tissues compared to pericarcinous tissues. We also demonstrate that the overexpression of HSP60 in kidney cancer cells which may restore mitochondrial activity suppresses cell growth and proliferation. We chose 293T, 786-O, 769-P and A549 cell lines as models to examine the effect of HSP60 silencing on tumor progression. 293T human embryonic kidney cells were characterized to have cancer stem cell-like features and are a useful model for studying cancer progression [37]. A549, a lung cancer cell line, was chosen because HSP60 was found to be downregulated in lung cancer tissues [14–15]. It was expected that HSP60 knockdown may induce similar effects on cellular processes in 293T, 786-O, 769-P and A549 cells. Indeed, the proliferation rates of HSP60-KN-293T, HSP60-KN-786-O, HSP60-KN-769-P and HSP60-KN-A549 cells were higher than control cells (Figure 2(B)-(E)) and the in vivo growth rates of these cells were even faster than control cells in nude mice (Figure 8 and Supplementary Figure S6), indicating that HSP60-knockdown promotes cell growth in 293T, 786-O, 769-P and A549 cells. Metabolomic analysis demonstrated that HSP60-KN-293T, HSP60-KN-786-O cells and HSP60-KN-769-P cells exhibited enhanced glycolysis and lactate production, the higher ROS level, and underwent EMT as compared to control cells. These characteristics resemble to tumorigenesis, reinforcing that HSP60 is a factor contributing to tumor progression in ccRCC.

An important finding in this study reveals that HSP60 knockdown decreases expressions of multiple subunits of respiratory complex I, leading to increased ROS production, which activates AMPK pathway. It is known that rotenone inhibits the respiratory complex I to generate ROS [38]. Our results are supported by the evidence that rotenone treatment also increases AMPK phosphorylation (Figure 4). As a result of AMPK activation, glycolysis is enhanced in HSP60-KN cells (Figure 5 and Supplementary Figure S5). Furthermore, the mitochondrial protein PDHA1 is down-regulated while MCT1 is up-regulated, which decreases the metabolite flux from glycolysis to TCA cycle and increases lactate production in HSP60-KN-293T (Figure 5). Similar results were observed in tumor samples from xenograft experiments (Supplementary Figure S7). All together, these results propose that HSP60 functions as a switcher for tuning glycolysis via enhanced ROS/AMPK signaling, leading to the Warburg-like metabolic phenotype in ccRCC cells. Importantly, our recent quantitative proteomics revealed that 19 subunits of the respiratory complex I were downregulated in ccRCC tissues as compared to pericarcinous tissues (Supplementary Figure S8), suggesting that the low level HSP60 expression also suppressed the complex I subunits expression in ccRCC.

In agreement with the early report [39], we found that HSP60-knockdown cells underwent the EMT process. Previous studies show that ROS enhances EMT in epidermal keratinocytes by secretion of TGF-β1 [40] and chronic oxidative stress leads to tumorigenesis and EMT in human renal epithelia cells [41]. Are the high levels of ROS promoting EMT in HSP60-KN cells? This is confirmed by the fact that the rotenone treatment induces EMT. Vimentin is ubiquitously expressed in normal mesenchymal cells and has been designated as a prognostic marker for ccRCC [42–44]. Our results indicate that HSP60 knockdown increases vimentin expression in 293T, 786-O and A549 cells, which is coincident with the upregulation of vimentin in ccRCC tissues as compared to the pericarcinous tissue (Supplementary Figure S9). We also noticed that HSP60 knockdown increased the cellular 2-HG production, suggesting the high level ROS in HSP60-KN cells attacked the Fe-S clusters and released Fe^2+^ ions that increased ADHFe1 expression, which converts 2-oxoglutarate to increase 2-HG production. This is in agreement with a recent metabolomic analysis of ccRCC tissues showing that 2-HG is elevated due to the down-regulation of L2HGDH [45]. These results evidently assert that ROS in HSP60-knockdown cells induces epigenetic changes via an increase of 2-HG that contributes to initiation and progression of ccRCC.

Taken together, as illustrated in Supplementary Figure S10, we demonstrate that the low level HSP60 expression is characteristic of ccRCC. HSP60 knockdown-mediated disruption of mitochondrial proteostasis and ROS overproduction drive metabolic reprogramming and enable cells to undergo EMT process, contributing to tumor progression of ccRCC.

## EXPERIMENTAL PROCEDURES

### Clinical human ccRCC specimens

18 pairs of ccRCC and pericarcinous tissue samples were collected after written informed consent from patients with ccRCC undergoing surgery at the Department of Urological Surgery of the General Hospital of PLA during 2013–2014 (Beijing, China). The group was composed of 14 men and 4 women with a mean age of 50 years (range 29–66 years) at the time of operation. Their clinical data and pathological features were presented in Supplementary Table S4, and confirmed by pathologists via post-operative pathology. The study was approved by the Scientific and Ethnic Committee of the General Hospital of PLA, Kidney cancer tissues were surgically removed from ccRCC patient while samples of pericarcinous tissues were obtained from the distal edge of the resection at least 6 cm from the tumor tissue. Part of the specimen was directly snap-frozen in liquid nitrogen, and stored at −80°C for extraction of total proteins. The other part of specimen was fixed in buffered formalin for 48 h, embedded in paraffin, and sectioned into 5 μm slides for immunohistochemistry (IHC) examination. Total proteins were extracted from paired tissues using 8 M Urea in PBS (pH 7.4) followed by western blotting.

### Cell lines

Human embryonic kidney 293T cell line (293T), lung cancer cell line A549 and ccRCC cell lines 786-O and 769-P were obtained from the Cell Bank of Type Culture Collection of Chinese Academy of Sciences (Shanghai, China). 293T cells were grown in DMEM media (Wisent, Montreal, QC). 786-O cells and 769-P cells were grown in 1640 media (Wisent, Montreal, QC). A549 cells were grown in F12K media. The culture media were supplemented with 10% fetal bovine serum (Wisent, Montreal, QC) and 1% penicillin/streptomycin (Wisent, Montreal, QC).

### Proteomics analysis

Proteomic analysis was carried out in biological triplicates as previously described [46]. Briefly, 100 μg of proteins extracted from cells was reduced by dithiothreitol and then alkylated with iodoacetamide. The protein samples were digested with trypsin for 20 h at room temperature. Tryptic peptides were desalted using Sep-Pak C18 cartridges, labeled with TMT reagents (Thermo, Pierce Biotechnology, Rockford, IL) according to the manufacture's instruction, and analyzed by LC-MS/MS.

### Establishment of stable HSP60 knockdown cell lines

The small hairpin RNA (shRNA) targeting HSP60 was chosen based on previous report [47]. The sequence was submitted for a BLAST search to ensure there was no existing homologous sequence. A scrambled non-silencing shRNA whose sequence is not found in the human genome database was used as negative control. Detailed shRNA sequences were listed in Supplementary Table S5. The HSP60-directed shRNAs and the scrambled non-silencing shRNA were cloned into pLL3.7 lentivirus vector. The lentivirus vectors were co-transfected in 293T cells with pMD2.G, pREV-Rev and pMDLg/pRRE using Lipofectamine 2000 (Invitrogen, Grand Island, NY). Supernatants were harvested after 48h, filtered through a 0.45 μM filter, and used to infect 293T, A549, 786-O and 769-P cells in the presence of 6 ug/ml of polybrene. Cells were sorted by a flow cytometer to generate the monoclonal stable cell line. The clone with intense and uniform GFP expression was selected and used in the present study.

### Establishment of stable HSP60 overexpression cell lines

The human HSPD1 gene was amplified from 293T cells and cloned to lentiviral vector pLVX-IRES-ZsGreen1. The lentivirus vectors were co-transfected in 293T cells with pLP1, pLP2 and pLP/VSVG using Lipofectamine 2000 (Invitrogen, Grand Island, NY). Supernatants were harvested after 48 h, filtered through a 0.45 μM filter, and used to infect 786-O cells in the presence of 6 ug/ml of polybrene. Cells were sorted by a flow cytometer to generate the monoclonal stable cell line. The clone with intense and uniform GFP expression was selected and used in the present study.

### Cell proliferation assay with CCK-8

Cells were seeded in 96-well plates with 2000 cells/well. Cell proliferation rate was determined with the Cell Counting Kit-8 (CCK-8) according to the manufacturer's instructions (Dojindo Laboratories, Kumamoto, Japan). Briefly, CCK-8 reagents were added into wells after cells grew for 0, 12 24, 36, 48, 72, and 84 h respectively. Absorbance at 450 nm was measured 2 h after CCK-8 addition.

### Survival rate of HSP60 knockdown cells treated with hydrogen peroxide

Effects of hydrogen peroxide on control and HSP60 knockdown cells were analyzed with the CCK-8 kit. Briefly, Cells were treated with hydrogen peroxide (250, 500, 750 and 1000 μM) in triplicates for 12 h. The CCK-8 reagent was added to treated cells and incubated at 37°C for 2 h. Optical density (OD) was measured at 450 nm with a microplate reader.

### Detection of cellular reactive oxygen species

The ROS in HSP60 knockdown cells was detected using CellROX® Deep Red Reagents (Invitrogen, Grand Island, NY) following manufacturer's instructions. Briefly, cells were stained with 5 μM CellROX® Deep Red Reagent by adding the probe to the complete medium and incubating the cells at 37°C for 30 min. The cells were then washed with PBS and analyzed on a BD FACSAria II Flow Cytometer (BD Biosciences, San Jose, CA).

### Western blotting

Cells were lysed in lysis buffer (20 mmol/L Tris-HCl pH 7.5, 150 mmol/L NaCl, 1% Triton X-100, 1% sodium pyrophosphate, Protease Inhibitor Cocktail) for 30 min on ice. The supernatant was collected after centrifugation at 14,000×g for 20 min at 4°C. Protein concentrations were determined using the BCA protein assay kit. Proteins were separated on the 12% SDS-PAGE gel and transferred onto a PVDF transfer membrane. Western blot analysis followed a standard procedure. NDUFA4, ENO2, MCT1 and β-catenin antibodies were obtained from Sigma (St Louis, MO). AMPKα, phospho-AMPKα (Thr172), PDHA1, E-cadherin, N-cadherin and β-actin were obtained from Cell Signaling Technology (Danvers, MA). The antibody for cytokeratin 18 was obtained from Millipore (Boston, MA). HIF1α antibody was obtained from Abcam (Cambridge, MA). Vimentin antibody was obtained from Proteintech (Chicago, IL). NDUFA5 and ADHFE1 antibodies were obtained from Pierce (Rockford, IL). HSP60 antibody was obtained from Stressgen (Victoria, BC).

### qPCR analysis

Total RNA was isolated using the SV Total RNA Isolation System and cDNA was synthesized using the GoScript^TM^ Reverse Transcription System (Promega, Fitchburg, WI) according to the manufacturer's instructions. All qPCRs were performed by using the Roche LightCycler 96 System with SYBR green incorporation (Promega, Fitchburg, WI). The primers for all genes used in this study are from primerbank (https://pga.mgh.harvard.edu/primerbank/index.html) and listed in Supplementary Table S6. 18SrRNA was used as internal control. The relative mRNA level was calculated using 2^−ΔΔCt^ method.

### Metabolomic analysis

The cells were washed twice with ice-cold PBS and extracted three times using 80% methanol (−80°C). The extracted metabolites were concentrated completely to dryness using a speedvac. The dried metabolites were dissolved in 80% methanol and used for LC-MS/MS analysis. To quantitatively measure the levels of metabolites extracted from control cells or HSP60 knockdown cells, two different mass spectrometry methods were employed. For targeted quantitative analysis, the TSQ Quantiva™ Triple Quadrupole Mass Spectrometer with positive/negative ion switching was used for targeted quantitation with selective reaction monitoring (SRM). The SRMs were constructed with parameters acquired through optimizing the collision induced fragmentation of purified standards of the given metabolites. The metabolite extracts were passed through a Synergi Hydro-RP column (2.0×100 mm, 2.5 μm, Phenomenex, Torrance, CA) that was interfaced with the mass spectrometer. For untargeted metabolomic profiling, the Q-Exactive Mass Spectrometer was used to carry out the LC-MS/MS analysis. Atlantis HILIC Silica column (2.1×100 mm, 3 μm, Waters, Milford, MA) and ACQUITY UPLC BEH Amide column (2.1×100 mm, 1.7 μm, Waters, Milford, MA) were used for positive and negative separation respectively. Metabolites were identified based on the retention time on the LC analysis and the accurate mass measured with <5 ppm mass accuracy. TraceFinder was used to identify the peaks and extract the quantitative information.

### Lipid analysis

5×10^6^ cells were suspended in 500 μl PBS, 3 ml of mixture of chloroform and methanol (V: V=2:1) was added. Samples were vortex for 30 s and centrifuged at 1000 rpm for 5 min. The lower chloroform layer was collected and dried by nitrogen. Samples were reconstituted with the mixture of chloroform and methanol (V: V=2:1) before LC-MS/MS analysis.

### Invasion assay

Invasion assay was performed using QCM 24-well cell invasion assay kit (Millipore, Boston, MA) according to the manufacturer's instructions. Briefly, cells were starved for 24 h prior to analysis. Cells were harvested using the 5 mL Harvesting Buffer per 100 mm dish and were then collected and re-suspended in 1-5 mL Quenching Medium. 1.25×10^5^ cells were seeded in the insert containing serum free media while the serum containing media were added to the lower chamber. Cells were incubated for 24 h at 37°C in a CO_2_ incubator. Cells that invaded through the ECMatrix-coated membrane were lysed in Lysis Buffer/Dye Solution and incubated for 15 min at room temperature. RFU values were read with a fluorescence plate reader using 480/520 nm filter set.

### Xenograft experiments

All animal studies were approved by the Animal Research Ethics Committee of the Tsinghua University. For xenograft experiments, 7×10^6^ HSP60-KN-293T or HSP60-KN-A549 and the control cells were harvested, washed twice with PBS, and resuspended in 150 μl PBS before being injected subcutaneously into 5-week-old female nude mice (Vital River Company, China). Tumor size was quantified by fluorescence imaging using IVIS in vivo imaging system (Perkin Elmer, Waltham, MA) at 14th days after injection. Tumor samples from the two animal groups were harvested at the end of imaging.

### Statistical analysis

Statistical analysis was carried out with GraphPad Prism 6.0 software by two sided unpaired *t* tests. P values of <0.05 were considered significant.

## SUPPLEMENTARY FIGURES AND TABLES






