# Supplementary material for: Downregulation of HSP60 disrupts mitochondrial proteostasis to promote tumorigenesis and progression in clear cell renal cell carcinoma
Source: Oncotarget. 2016 May 26;7(25):38822–34. doi: 10.18632/oncotarget.9615 (PMC5122432; doi:10.18632/oncotarget.9615)
Supplement: Supplementary file 1 [file oncotarget-07-38822-s001.pdf]

Downregulation of HSP60 disrupts mitochondrial proteostasis to promote tumorigenesis and progression in clear cell renal cell carcinoma

SUPPLEMENTARY FIGURES AND TABLES

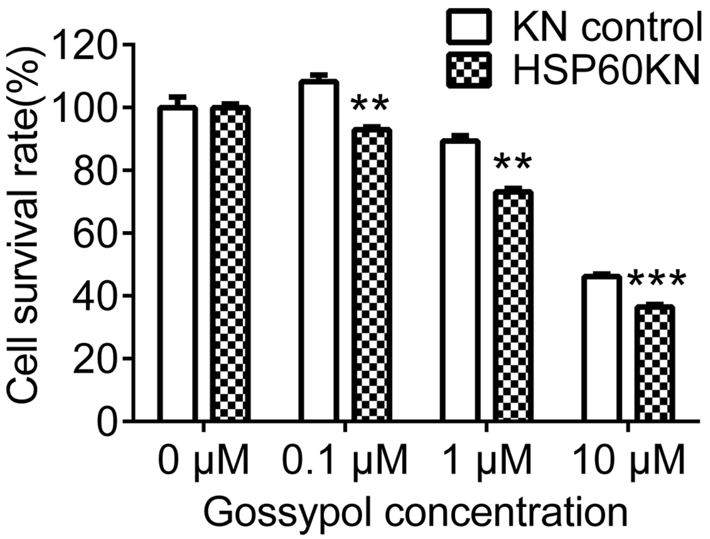

Supplementary Figure S1: Survival rate of HSP60-KN-293T and the control cells treated with different concentration of gossypol. The survival rate shows significant differences between HSP60-KN-293T and control cells with the treatment of gossypol. Data were analyzed using student's t test. *p<0.05, **p<0.01 and *** p< 0.001. *p < 0.05 is considered statistically significant. Error bars represent ±SEM.

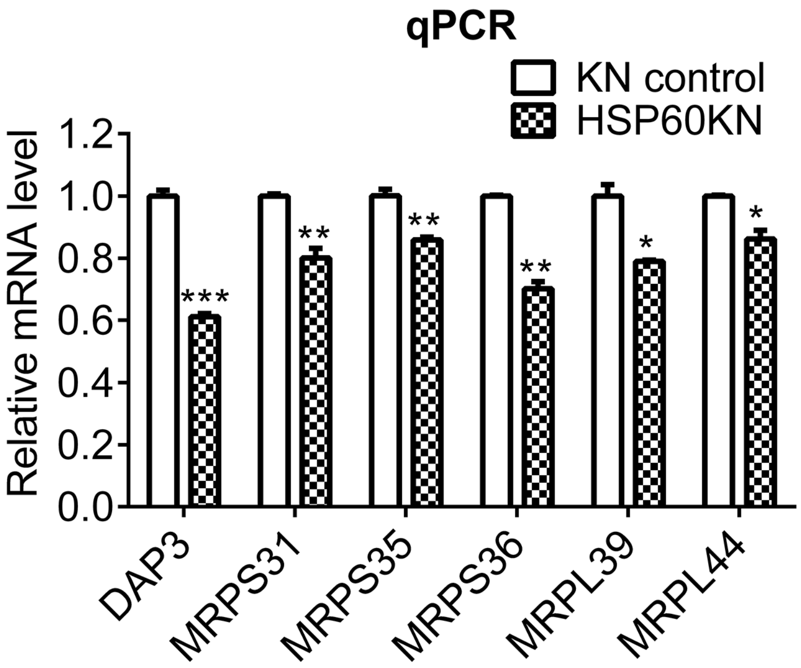

Supplementary Figure S2: The mRNA levels of ribosomal proteins are down regulated in HSP60-KN-293T cells. Data were analyzed using student's t test. *p<0.05, **p<0.01 and *** p< 0.001. *p < 0.05 is considered statistically significant. Error bars represent ±SEM.

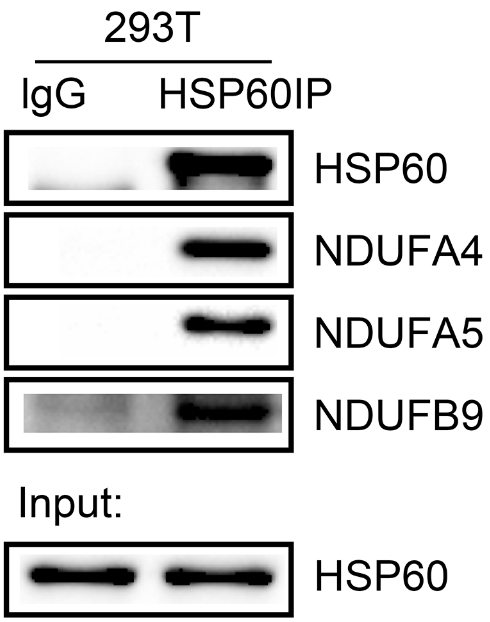

Supplementary Figure S3: Western blot images of NDUFA4, NDUFA5 and NDUFB9 in the immunoprecipitated HSP60 complex. IP was performed with mouse anti-HSP60 antibody followed by immunoblotting with rabbit anti- NDUFA4, NDUFA5 and NDUFB9 antibody. Mouse IgG was used as negative control.

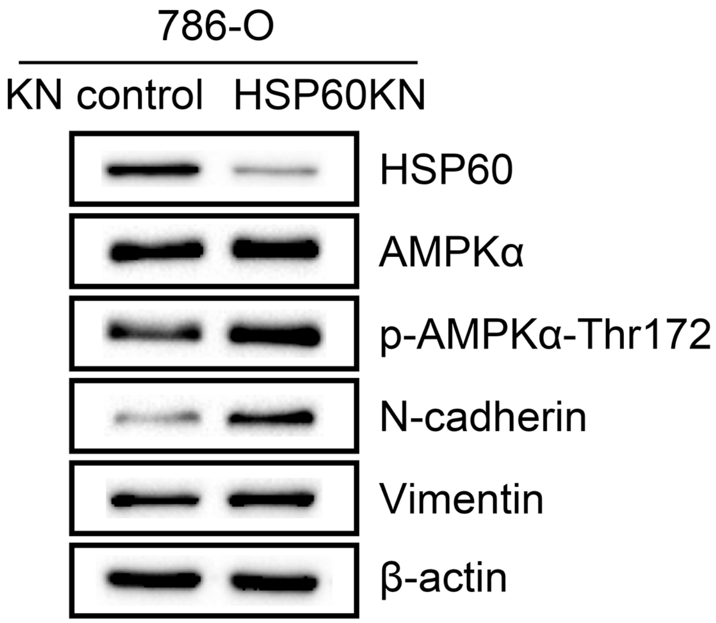

Supplementary Figure S4: HSP60 knockdown in 786-O cells activates AMPK pathway and drives cell to undergo EMT process. Western blot images of expression levels of HSP60, AMPK, N-cadherin and vimentin in the control cells and HSP60-KN-786-O cells.

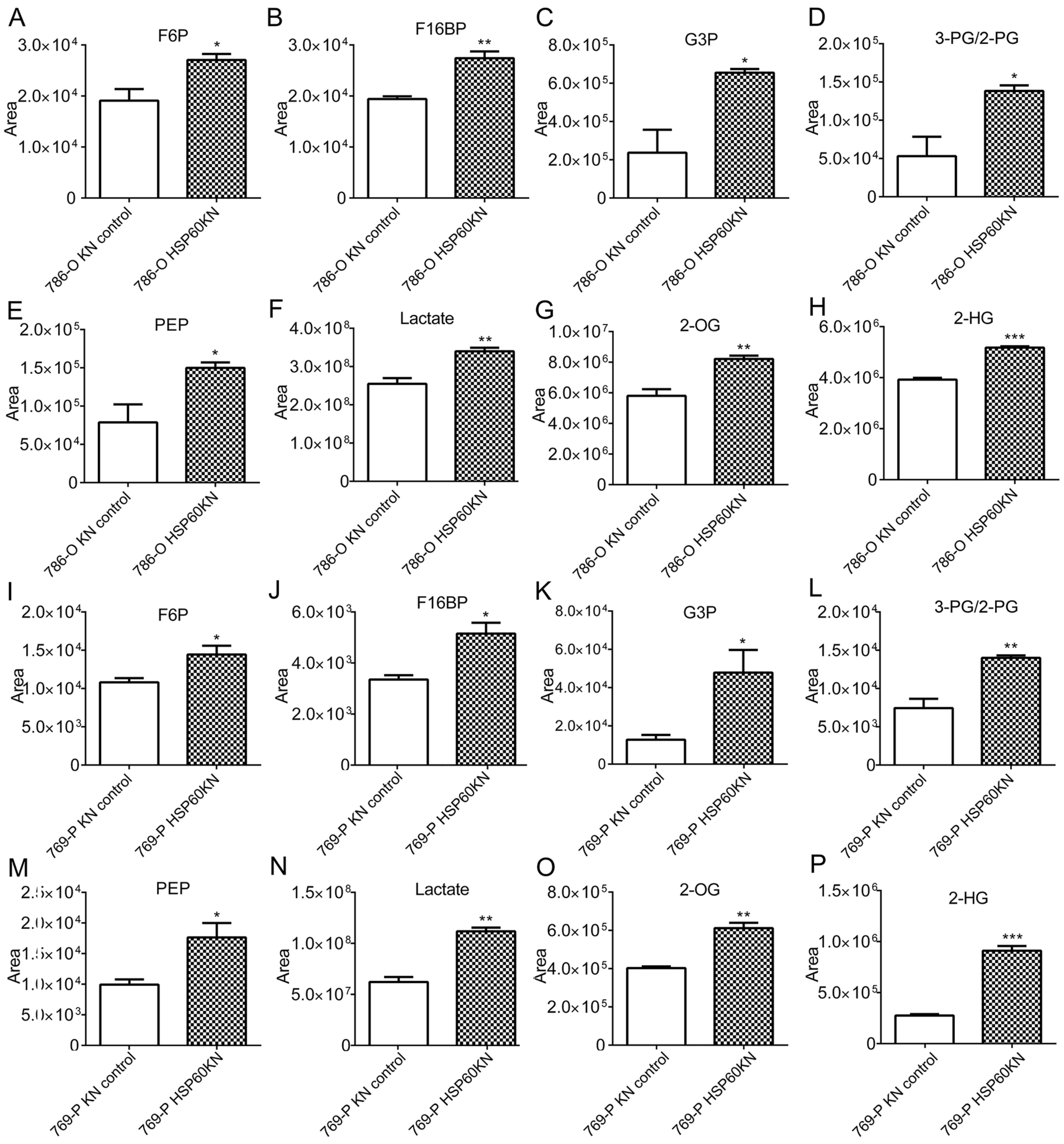

Supplementary Figure S5: HSP60 knockdown enhances glycolysis in 786-O cells and 769-P cells. A-H. Graphical representations of relative concentrations of F6P; F16BP; G3P; 3-PG/2-PG; PEP ; lactate ; 2-OG and 2-HG in HSP60-KN-786-O cells compared to the control cells. **I-P.** Graphical representations of relative concentrations of F6P; F16BP; G3P; 3-PG/2-PG; PEP ; lactate ; 2-OG and 2-HG in HSP60-KN-769-P cells compared to the control cells. Data were analyzed using student's t test. *$p<0.05$, **$p<0.01$ and *** $p< 0.001$. *$p < 0.05$ is considered statistically significant. Error bars represent ±SEM.

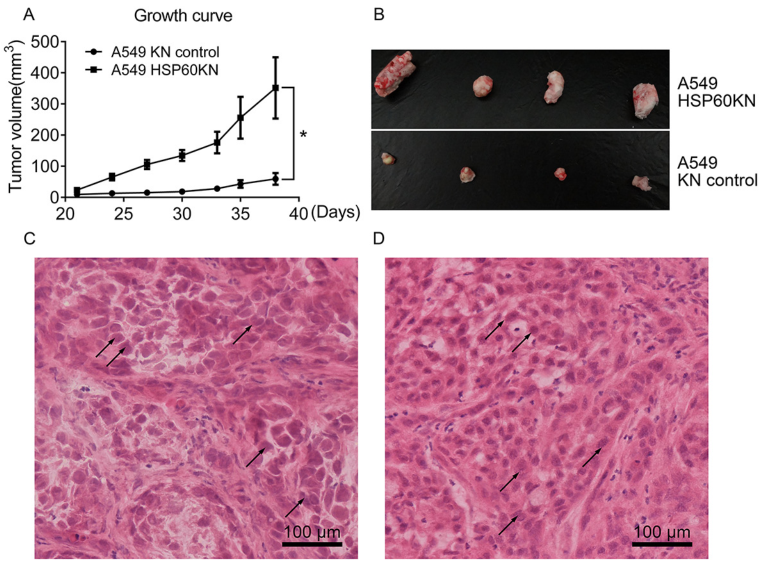

Supplementary Figure S6: HSP60 knockdown promotes in vivo tumor growth of HSP60-KN-A549 cells in nude mice. **A.** Growth curves of tumors produced by HSP60-KN-A549 and the control cells which were injected subcutaneously into nude mice. The tumor volumes (mm^3) were measured using digital calipers every 3 days after injection and calculated using the formula: π/6× length (mm) × $width^2$ (mm). Data were analyzed using student's t test. *p<0.05, **p<0.01 and *** p< 0.001. Error bars represent ±SEM. **B.** Images of tumor samples harvested from mice injected with HSP60-KN-A549 and the control groups, respectively; **C.** and **D.** images of the H&E staining of tissues from mice injected with the control cells (C) and HSP60-KN-A549 cells (D), arrows indicate tumor cells.

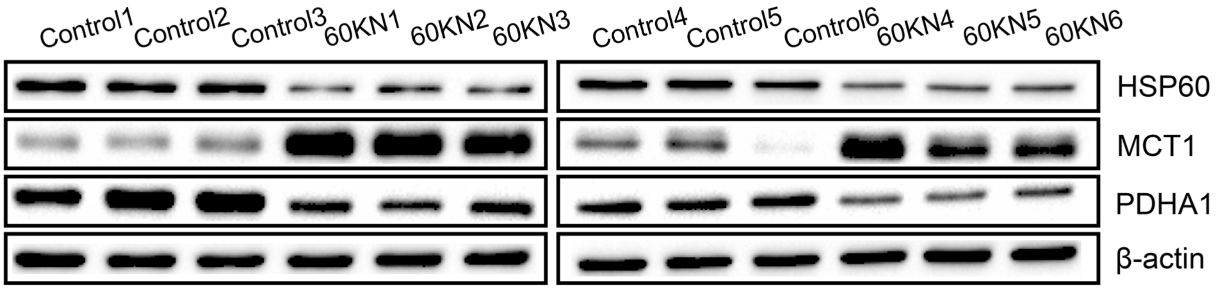

Supplementary Figure S7: Western blotting analysis of HSP60, MCT and PDHA1 in tumor samples from 293T xenograft experiments.

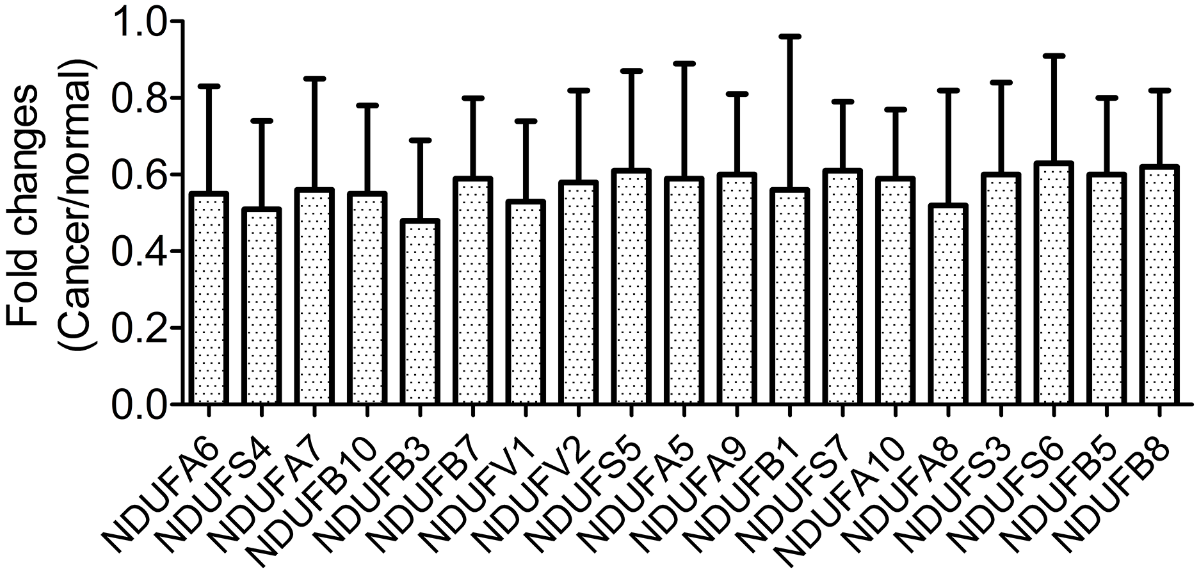

Supplementary Figure S8: Quantitative proteomics revealed that 19 subunits of the respiratory complex I were downregulated in ccRRC tissues as compared to pericarcinous tissues.

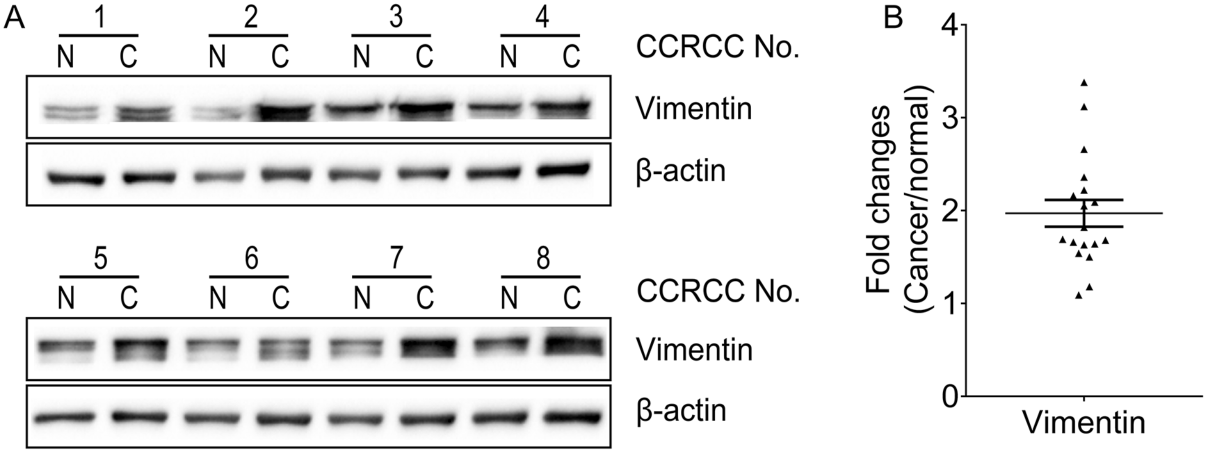

Supplementary Figure S9: Upregulation of vimentin in ccRCC compared to associated pericarcinous tissues. A. Western blotting images of the expression levels of vimentin in 8 of 18 paired ccRCC lesions and associated pericarcinous tissues; **B.** the gray scale analysis of vimentin in 18 paired ccRCC lesions compared to associated pericarcinous tissues.

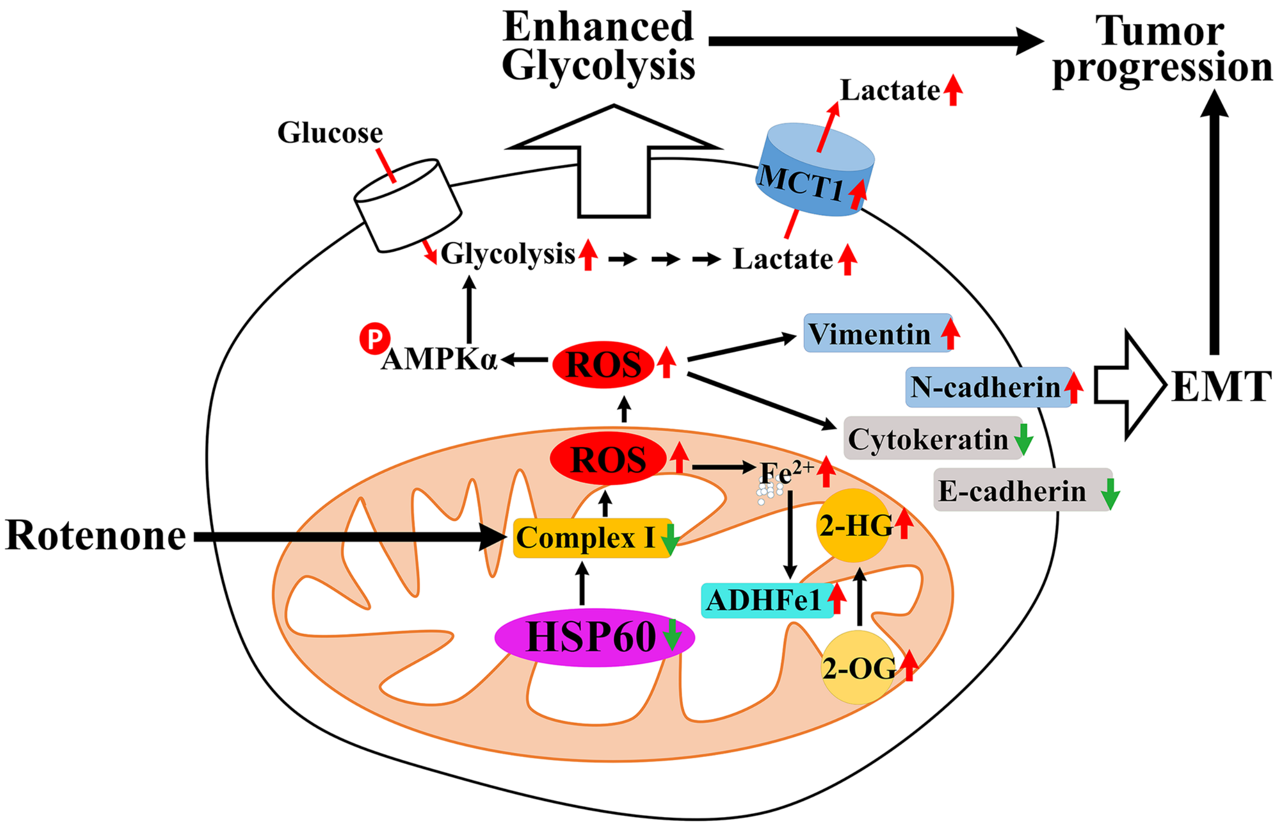

Supplementary Figure S10: A graphic illustration of effects of HSP60 silencing on cellular processes and tumor progression in ccRCC.

Supplementary Table S1: The list of up-regulated proteins in HSP60-KN-293T cells compared to the control cells

Accession Number	Description	Score	Sequence Coverage (%)	Unique Peptides	Fold Change (HSP60-KN/ control)	MW (kDa)
Q709F0	Acyl-CoA dehydrogenase family member 11	72.9	20.3	15	1.6	87.2
P06727	Apolipoprotein A-IV	20.6	3.8	1	2.4	45.4
Q8NDZ4	Deleted in autism protein 1	5.6	5.1	2	1.5	49.5
P17661	Desmin	476.2	10.6	1	2.5	53.5
Q16401-2	Isoform 2 of 26S proteasome non-ATPase regulatory subunit 5	36	18.4	8	2.1	51.3
Q9P1U1-2	Isoform 2 of Actin-related protein 3B	33.5	16.3	1	1.8	38
P62633-2	Isoform 2 of Cellular nucleic acid-binding protein	196.3	72.9	1	1.5	18.7
P09104-2	Isoform 2 of Gamma-enolase	1250.7	41.9	8	1.5	42.7
Q8NEM0-2	Isoform 2 of Microcephalin	10.8	6.2	3	7.8	62.3
Q5ZPR3-3	Isoform 3 of CD276 antigen	10.3	8.5	2	1.6	52.7
Q14195-2	Isoform LCRMP-4 of Dihydropyrimidinase-related protein 3	110.4	29.1	13	1.8	73.9
Q14533	Keratin, type II cuticular Hb1	14.3	4.6	1	1.5	54.9
G3V2C4	Mirror-image polydactyly gene 1 protein (Fragment)	6.4	17.2	1	1.9	15
P53985	Monocarboxylate transporter 1	35.2	6.6	3	1.5	53.9
Q14697	Neutral alpha-glucosidase AB	342.9	44.7	1	2	106.8
E9PI87	Oxidoreductase HTATIP2	16.5	29.6	6	1.9	22
P0CG39	POTE ankyrin domain family member J	1520.4	6.7	1	1.7	117.3
Q9BZQ8	Protein Niban	11.6	5.1	5	1.6	103.1
E5RGX5	Stathmin	237.3	24.4	1	1.6	19.6
Q92922	SWI/SNF complex subunit SMARCC1	375.3	33.7	28	1.5	122.8
P08670	Vimentin	785.2	66.1	29	2.7	53.6
Q96H79	Zinc finger CCCH-type antiviral protein 1-like	115.9	48.3	11	1.5	32.9
H0Y362	Zinc transporter 7 (Fragment)	10.7	17.2	1	1.8	9.9

Supplementary Table S2: The list of down-regulated proteins in HSP60-KN-293T cells compared to the control cells.

See Supplementary File 1

Supplementary Table S3: The list of subunits of complex I in the immunoprecipitated HSP60 complex as determined by the TMT ratios.

See Supplementary File 2

Supplementary Table S4: Clinical Characteristics of Patients with ccRCC

NO.	Age	Sex	Histopathology type	Laterality	Tumor Size (cm)	Grading	Staging
1	52	Male	Clear cell carcinoma	Right	3.5×3.5×3	II	T1a
2	46	Male	Clear cell carcinoma	Right	2.7×2.3×0.8	II	T1a
3	47	Male	Clear cell carcinoma	Right	6.5×6×5	II	T1b
4	58	Male	Clear cell carcinoma	Right	2.5×2×2	I-II	T1a
5	45	Male	Clear cell carcinoma	Right	5.5×4×4	II	T1b
6	62	Female	Clear cell carcinoma	Right	5×5×2.5	I-II	T1a
7	65	Male	Clear cell carcinoma	Left	2×1.8×1	II	T3a
8	61	Male	Clear cell carcinoma	Left	5.5×4.5×3	II	T1a
9	60	Male	Clear cell carcinoma	Left	7×4.5×3	II	T1b
10	51	Male	Clear cell carcinoma	Left	7×6×5.5	II	T1b
11	36	Female	Clear cell carcinoma	Right	1.5×1.5×1.5	I-II	T1b
12	45	Male	Clear cell carcinoma	Left	4×4×3	I-II	T1a
13	39	Female	Clear cell carcinoma	Left	5.5×5×3	I	T1a
14	29	Female	Clear cell carcinoma	Right	3×2.8×2	II	T1b
15	58	Male	Clear cell carcinoma	Left	3.5×3.5×2.5	II	T1a
16	42	Male	Clear cell carcinoma	Left	5×4.5×3.5	II-III	T1a
17	46	Male	Clear cell carcinoma	Right	6×5.5×5.5	I	T1b
18	66	Male	Clear cell carcinoma	Left	6×5.5×5.5	II	T1b

Supplementary Table S5: The sequences of control shRNA and HSP60-directed shRNA

Name	Sequence (5'- 3')
shRNA-HSP60	TGCAGGGTTTGGTGACAATAGAATTCAAGAGATTCTATTGTCACCAAACCCTGTTTTTTC
shRNA-non-silencing control	TGTTCTCCGAACGTGTCACGTTTCAAGAGAACGTGACACGTTCGGAGAATTTTTTC

Supplementary Table S6: The sequences of qPCR primers

Gene	Primer name	Sequence (5'-3')
DAP3	Forward primer	TCCAGCTACAACAAACAGCG
	Reverse primer	CTCACCCGTGTTATGCCCTG
MRPS31	Forward primer	AGAGTCTCGACGTTCCTACCT
	Reverse primer	TGAGTAGCATAATCGCAGCCG
MRPS35	Forward primer	GGAAAGAACACCCGGAAATGA
	Reverse primer	GTGCTGCAACTGGGTAAACAC
MRPS36	Forward primer	GCGTCTGCTAGTAGGGTCG
	Reverse primer	AAGAGTGAGATGGTAGCCCTG
MRPL39	Forward primer	CCGACAGAATTGACAGAAATGCG
	Reverse primer	CTTCTCAGTTCGGGGAGTTAATG
MRPL44	Forward primer	TCCGGGCTGGTAAGATTGC
	Reverse primer	CGCGGAATCCCTTCTTCAC
18S rRNA	Forward primer	GTAACCCGTTGAACCCCATT
	Reverse primer	CCATCCAATCGGTAGTAGCG
