# Supplementary material for: Downregulation of HSP60 disrupts mitochondrial proteostasis to promote tumorigenesis and progression in clear cell renal cell carcinoma
Source: Oncotarget. 2016 May 26;7(25):38822–34. doi: 10.18632/oncotarget.9615 (PMC5122432; doi:10.18632/oncotarget.9615)
Supplement: Supplementary file 3 [file oncotarget-07-38822-s003.docx]

Supplementary Table S3. The list of subunits of complex I in the immunoprecipitated HSP60 complex as determined by the TMT ratios.

| Accession Number | Description | Score | Sequence | Unique Peptides | Fold Change | MW (kDa) |
| --- | --- | --- | --- | --- | --- | --- |
|  |  |  | Coverage (%) |  | (HSP60IP/IgG) |  |
| O75251 | NADH dehydrogenase [ubiquinone] iron-sulfur protein 7, mitochondrial | 15.6 | 19.3 | 5 | 4.2 | 23.5 |
| O00483 | NADH dehydrogenase [ubiquinone] 1 alpha subcomplex subunit 4 | 31.1 | 45.7 | 4 | 5.7 | 9.4 |
| Q7L592 | NADH dehydrogenase [ubiquinone] complex I, assembly factor 7 | 49.0 | 31.5 | 12 | 6.8 | 49.2 |
| F5H7V7 | NADH dehydrogenase [ubiquinone] 1 alpha subcomplex subunit 8 | 7.2 | 21.9 | 3 | 4.5 | 14.9 |
| Q16795 | NADH dehydrogenase [ubiquinone] 1 alpha subcomplex subunit 9, mitochondrial | 55.1 | 31.3 | 11 | 4.8 | 42.5 |
| S4R3I5 | NADH dehydrogenase (Ubiquinone) 1 alpha subcomplex, 3, 9kDa, isoform CRA_b | 13.3 | 26.8 | 1 | 4.1 | 4.6 |
| K7EQ77 | NADH dehydrogenase [ubiquinone] 1 alpha subcomplex subunit 11 | 17.7 | 39.2 | 3 | 4.3 | 12.7 |
| P28331 | NADH-ubiquinone oxidoreductase 75 kDa subunit, mitochondrial | 348.3 | 60.4 | 37 | 3.6 | 79.4 |
| Q9BU61 | NADH dehydrogenase [ubiquinone] 1 alpha subcomplex assembly factor 3 | 25.6 | 37.5 | 5 | 3.5 | 20.3 |
| O95298 | NADH dehydrogenase [ubiquinone] 1 subunit C2 | 3.3 | 15.1 | 2 | 2.9 | 14.2 |
| H3BPJ9 | NADH dehydrogenase [ubiquinone] 1 beta subcomplex subunit 10 | 45.6 | 46.6 | 7 | 2.9 | 19.2 |
| Q9P032 | NADH dehydrogenase [ubiquinone] 1 alpha subcomplex assembly factor 4 | 32.9 | 39.4 | 8 | 2.8 | 20.3 |
| Q9Y6M9 | NADH dehydrogenase [ubiquinone] 1 beta subcomplex subunit 9 | 32.4 | 35.8 | 5 | 3.1 | 21.8 |
| Q16718 | NADH dehydrogenase [ubiquinone] 1 alpha subcomplex subunit 5 | 19.2 | 43.1 | 4 | 2.4 | 13.5 |
| E9PKH6 | NADH dehydrogenase [ubiquinone] iron-sulfur protein 8, mitochondrial (Fragment) | 43.7 | 46.4 | 6 | 3.3 | 15.9 |
| O95299 | NADH dehydrogenase [ubiquinone] 1 alpha subcomplex subunit 10, mitochondrial | 63.1 | 22.5 | 8 | 5.1 | 40.7 |
| O43678 | NADH dehydrogenase [ubiquinone] 1 alpha subcomplex subunit 2 | 23.5 | 48.5 | 4 | 2.6 | 10.9 |
| Q9P0J0 | NADH dehydrogenase [ubiquinone] 1 alpha subcomplex subunit 13 | 39.1 | 51.4 | 8 | 2.4 | 16.7 |
| O75380 | NADH dehydrogenase [ubiquinone] iron-sulfur protein 6, mitochondrial | 11.6 | 33.1 | 3 | 2.1 | 13.7 |
| O75438 | NADH dehydrogenase [ubiquinone] 1 beta subcomplex subunit 1 | 6.0 | 19.0 | 1 | 2.2 | 7.0 |
| Q330K2-2 | Isoform 2 of NADH dehydrogenase (ubiquinone) complex I, assembly factor 6 | 4.7 | 9.3 | 3 | 7.6 | 32.8 |
| O95168-2 | Isoform 2 of NADH dehydrogenase [ubiquinone] 1 beta subcomplex subunit 4 | 22.3 | 44.2 | 5 | 2.9 | 14.0 |
| O95139-2 | Isoform 2 of NADH dehydrogenase [ubiquinone] 1 beta subcomplex subunit 6 | 17.4 | 22.1 | 4 | 6.4 | 13.7 |
| O75306-2 | Isoform 2 of NADH dehydrogenase [ubiquinone] iron-sulfur protein 2, mitochondrial | 207.6 | 45.3 | 18 | 6.0 | 51.8 |
